# Knowledge, attitude, and practice towards Hepatitis B infection among nurses and midwives in two maternity hospitals in Khartoum, Sudan

**DOI:** 10.1186/s12889-019-7982-8

**Published:** 2019-11-29

**Authors:** Sanaa Mohammed-elbager Mahmoud Mursy, Sagad Omer Obeid Mohamed

**Affiliations:** Faculty of Medicine, University of Khartoum, Qasr Street, P.O. Box 11111, Khartoum, Sudan

**Keywords:** Hepatitis B, Sudan, KAP

## Abstract

**Background:**

Hepatitis B virus (HBV) infection creates a global health burden with significant morbidity and mortality. Healthcare workers, including nurses and midwives, are at higher risk of acquiring the disease. While health-related behaviours are affected by different aspects of knowledge, attitude, and practices (KAP), there are few studies examining the KAP level of healthcare workers towards HBV infection in Sudan. The purpose of this study was to examine the KAP level of nurses and midwives towards HBV virus infection in Khartoum, Sudan.

**Methods:**

A cross-sectional descriptive hospital-based study was conducted in two public maternity hospitals (Saudi and Saad Abul-Eleella hospitals) in Khartoum state of, Sudan. A pre-tested structured questionnaire was constructed and implemented to examine KAP towards HBV infection. Statistical Package for Social Sciences (SPSS) version of 21 was utilized to conduct statistical analysis and examine the data at hand. Chi-square test was used implemented to determine the relationship between categorical variables.

**Results:**

A total of 110 nurses and midwives from the both hospitals participated in this study. More than half of the respondents (58.2%) had an average level of knowledge, two-third of the respondents had a safe practice, and the majority of the respondents had a favourable attitude towards HBV preventive measures. Approximately half of the participants (51.8%) had a history of needle stick injuries. Half of the participants had inaccurate concepts about post exposure prophylaxis to HBV infection, while more than half of the nurses and midwives didn’t complete the vaccination schedule for HBV.

**Conclusion:**

Most of the nurses and midwives in Saudi and Saad Abul-Eleella hospitals were aware of HBV infection. However, a significant proportion of the participants lack the requisite knowledge about post exposure management. The study revealed a low level of HBV vaccination coverage rate and a high rate of needle stick injuries. Further strategies for preventing workplace exposure, training programs on HBV infection, including post exposure prophylaxis, and increasing vaccination coverage rate of all HCWS are highly recommended.

## Background

Hepatitis B virus (HBV) infection creates a global health burden with significant morbidity and mortality from both acute infection and chronic complications, including chronic hepatitis, cirrhosis, and hepatocellular carcinoma [1–5]. The number of patients with positive hepatitis B surface antigen (HBsAg) worldwide increased from 223 million in 1990 to 240 million in 2005 [6]. Several studies have provided estimates of the prevalence of HBV infection among several Sudanese populations [3, 7–9]. A recent systematic review and meta-analysis including 14 studies with 5848 participants have been conducted in Sudan, revealing that HBV seroprevalence rates ranged from 5.1 to 26.8% with an overall pooled prevalence of 12.1%. According to study findings, Khartoum State had the highest prevalence of HBV infection in Sudan with a proportion of 12.7% [1]. Likewise, this rate is comparable to other African countries such as Burundi (15.6%), Central African Republic (14%), while it is higher than Nigeria (5%) and Ethiopia (7%) [2].

Risk factors for HBV infection include transfusion of infectious blood and mother to child transmission (MTCT) of the virus [2, 3]. Pregnancy is well tolerated by women with chronic hepatitis B infection, but postpartum reconstitution of the immune system is associated with an elevated level of liver enzymes and active hepatitis [10]. MTCT of HBV can occur at three stages of pregnancy: intrauterine, intrapartum or postpartum, and the risk for MTCT of HBV increases with high maternal viral load, positive HBeAg status, preterm labour, prolonged labour and failure of immunoprophylaxis in siblings [10, 11]. The current standard of care for the prevention of MTCT of HBV infection includes an active and passive immunoprophylaxis with HBV immunoglobulin and vaccination administered immediately after birth to neonates of HBsAg positive mothers, and referral of mothers to hepatology clinics for assessment and follow up [11, 12].

Healthcare workers (HCWs), including nurses and midwives, had a higher risk of exposure and acquired the disease if the personal protective measures were not appropriately used [13, 14]. Onwards HBV transmission could be related to lack of awareness about HBV prevalence and occupational safety measures such as vaccination against HBV, post-exposure prophylaxis (PEP), training and adopting safer working practices [1, 9, 10]. Handling sharps and needle stick injuries (NSI) represent major risks for unvaccinated HCWSs and involve potential exposure to several pathogens, including HBV [14, 15].

In the study setting, nurses and midwives have an important role in the care of people with HBV infection. They provide support during treatment and education about the nature of the disease, diagnosis, prevention, and timely administration of immunoglobulins. To implement an effective management plan, nurses and midwives should have a basic understanding of the disease and its various implications for the patients [16].

Evidence suggests that health-related behaviours are affected by knowledge, attitude, and practice (KAP) [17, 18]. However, there are few studies examining the KAP level of HCWSs towards HBV infection in Sudan [3, 15]. KAP surveys have been widely implemented in public health research and have been reported to be the most commonly used study tools in health-seeking behaviour research [19]. KAP studies have been used to collect information on what the participants know, believe and do in relation to a particular topic [17–20]. The *knowledge* refers to the understanding of any given topic [21, 22]. *Attitude* refers to their feelings towards this subject, preconceived ideas that they may have towards it, intention to a particular behaviour and inclination to react in a certain way to a certain situation [19, 21, 22]. *Practice* refers to the ways in which they do and demonstrate their knowledge and attitude through their actions [22].

The purpose of this study was to examine the KAP towards HBV infection among nurses and midwives, which could help to inform the strategies for prevention and control of HBV infection in Khartoum, Sudan.

## Methods

### Study design and setting

A descriptive hospital-based cross-sectional study was conducted in Saudi and Saad Abul-Ella hospitals in Khartoum state, Sudan between 18 August and 2 September 2016. These settings were chosen because they are public health, tertiary healthcare facilities providing specialized clinical inpatient and outpatient services for a great number of the Khartoum state population. These two hospitals are popular health facilities specialized in obstetrics and gynaecology services and are main referral hospitals in Khartoum. Khartoum state is the most populous state in Sudan with a population of more than 5 million in the 2008 census. The state has experienced rapid urbanization during the last decades, and the large part of the state’s population has reported that their region of origin is outside Khartoum. The state is divided into seven localities. The distribution of public and private hospitals in these localities is made according to the population density.

The total number of nurses and midwives working at the two participating hospitals are 150 (53 nurses and 30 midwives in Saudi hospital, and 57 nurses and 10 midwives in Saad Abul-Ella hospital). The inclusion criteria for this study were nurses and midwives working at these two hospitals with the exclusion of those refusing to participate in the study.

### Data collection and analysis

The study implemented a 31-item structured questionnaire consisting of four sections (Additional file 1) to examine the KAP level of nurses and midwives towards HBV infection. The data were collected from HCWs who met eligibility criteria. The structured questionnaire consisted of six questions for the demographic information, 15 questions for knowledge, four questions for attitude, and six questions for practice. When a respondent has no history of needle stick injury, she is supposed to answer three of the practice questions and left the others.

The main purpose of the knowledge questions is to measure the basic knowledge about aetiology, natural history, modes of transmission, complications, and PEP to HBV. The response set of these questions was set as “yes” and “no” choices. Respondents with favourable attitude are those who answered questions which indicated they believe that instruments sterilization, wearing gloves, and vaccination are important to prevent HBV transmission recommend PEP for those who had been exposed to HBV. The questions regarding practice section aimed to measure whether the participants received HBV vaccine, sterilize instruments, and wear gloves. When the participants had NSI, they were asked whether they used water and soap when they washed their hands, sterilized the wound site, and received PEP. The response set of practice questions was set as “yes” if a participant reported that she performed the practice and “no” if she did not.

A scoring system was generated, and participants were given a score on each correct answer provided. The overall KAP score was determined based on the sum of correct answers to the 15 knowledge-based questions, four attitude-based questions and six practice-based questions (or three practice-based questions if the participant had no history of NSI). A correct response to each question received one point. Median was used to determine the cut-off point for each section of KAP; equal to or more than 12 out of 15 were considered as average knowledge, equal to or more than three out of 4 were considered as favourable attitude, and equal to or more than five out of six were considered as safe practice, and when there is no history of NSI, total of three were considered as safe practice. The questionnaire was constructed based on experts’ advice (Department of Community Medicine, Faculty of Medicine, University of Khartoum). The questionnaire was administered to 14 respondents and pretested for its clarity and acceptability, and data from pilot study were not considered in the final analysis. The Statistical Package for Social Sciences (SPSS) software version of 21 was utilized to analyse the data at hand. Descriptive statistics of SPSS provided frequency tables and the distribution of the variables. A Chi-square test was conducted to determine the relationship between the independent categorical variables and the main outcomes of the study (knowledge, attitude, and practice related to HBV infection). The *p*-value of ≤ .05 was set as the significance level of the study.

### Ethical consideration and consent

The institutional review board of Faculty of Medicine, University of Khartoum granted permission for conducting this research before study initiation, and a permission was also granted by the general directors of Al Saud and Saad Abul-Ella hospitals, Khartoum, Sudan. Ethical approval was obtained from the State Ministry of Health in Khartoum state, Sudan. After providing a clear explanation, each respondent’s informed written consent was obtained before participation. Confidentiality of the study participants was maintained.

## Results

A total of 110 nurses and midwives from Saudi hospital and Saad Abul-Ella hospital participated in this study with the number of 64 (58.2%) and 46 (41.8%), respectively. Among the 110 participants, 80 (72.7%) of them were nurses and 30 (27.3%) of them were midwives. The overall response rate was 73.3% among the 110 participants. The response rate was 72.7% among the 80 eligible nurses working at the both hospitals while it was 75% among the 40 eligible midwives working at the both hospitals. All of the respondents were females with a mean age of 32.9 ± 11.8 years. A clear majority of the participants (80, 72.7%) had an undergraduate degree or above, while 30 participants (27.3%) had an undergraduate degree. More than half of the participants (65, 59%) had less than 2 years of work experience and 45 (40.9%) of them had more than 2 years of work experience. The demographic characteristics of the participants were presented in Table 1.

**Table 1:** Demographic characteristics of the respondents in Saudi and Saad Abul-Ella hospitals

Demographic variables		Number	Percent %
Age Group	21–30 year	72	65.5%
	31–40 year	12	10.9%
	41–50 year	13	11.8%
	More than 50	13	11.8%
Hospital	Saudi	64	58.2%
	Saad Abul-Ella	46	41.8%
Occupation	Nurse	80	72.7%
	Midwife	30	27.3%
Educational level	below university	30	27.3%
	university or above	80	72.7%
Experience years	< 2 years	65	59.1%
	> 2 years	45	40.9%

### Assessment of knowledge towards HBV

Status of knowledge among the respondents was summarized in Table 2. The study findings revealed that the percentage of respondents with average knowledge regarding HBV is 58.2%. According to study results, HBV was recognized as a viral infection by overwhelming majority of the respondents (90.9%). The majority of the participants reported that HBV could cause acute and chronic infection with a proportion of 72.7 and 64.5%, respectively. Liver cirrhosis, hepatocellular carcinoma, and death were recognized as possible complications by the participants with a proportion of 82.7, 52.7, and 81.8%, respectively. Knowledge about PEP was average only in half of the respondents (50%).

**Table 2:** Knowledge, attitude, and practice of nurses & midwives regarding HBV infection in Saudi and Saad Abul-Ella hospitals

	Number (correct answer)	Percent (correct answer)
HBV knowledge items		
There are several types of hepatitis	104	94.5%
Hepatitis B is a viral infection	100	90.9%
The virus can cause acute hepatitis	71	64.5%
The virus can cause chronic hepatitis	80	72.7%
The virus can cause liver cirrhosis	91	82.7%
The virus can cause hepatocellular carcinoma	58	52.7%
The infection can cause death	90	81.8%
The virus doesn’t cause peptic ulcer	79	71.8%
The virus can be transmitted by transfusion of infectious blood.	105	95.5%
The virus can be transmitted by sexual intercourse	86	78.2%
The virus can be transmitted by MTCT.	83	75.5%
The virus isn’t transmitted by with all kinds of contact with infected persons.	71	64.5%
The virus can be transmitted by NSI	96	87.3%
The virus is not transmitted through air	88	80.0%
Post-exposure prophylaxis includes IG & vaccination	55	50.0%
HBV attitude items		
Do you believe that instrument sterilization is important to prevent transmission?	71	64.5%
Do you believe that wearing gloves is important to prevent transmission?	80	72.7%
Do you believe that vaccination could prevent transmission?	91	82.7%
Do you recommend PEP for those who had been exposed to HBV?	58	52.7%
HBV practice items		
They always Sterilize instruments	99	90.0%
They always wear gloves	100	90.9%
Completed the HBV vaccination schedule	45	41.0%
History of NSI	57	51.8%
Washing hands with water & soap after NSI	47	82.5%
Sterilized the wound site after NSI	51	89.5%
Check if the patient have a blood-borne disease after NSI	39	68.9%

*IG* immunoglobulins, *MTCT* mother to child transmission, *PEP* Post exposure prophylaxis

### Assessment of attitude towards HBV

The great majority of the respondents (86.4%) showed a favourable attitude towards the preventive measures of HBV infection (instruments sterilization, wearing gloves, HBV vaccination, and PEP). More than half of the participants (64.5%) believed that instruments sterilization is important to prevent transmission, and 72.7% of them think that wearing gloves is important to prevent transmission. A vast majority of the participants (82.7%) reported that vaccination is an important protective measure. Only 52.7% of the participants said that they recommend PEP immediately after exposure to infection (Table 2).

### Assessment of practice towards HBV

Among the respondents, 65.5% of them were considered to have a safe practice regarding HBV infection. According to the study findings, a vast majority of the participants always used sterilized instruments (90%) and wore gloves (90.9%). Near half of the participants (51.8%) had a history of NSI. Among the participants, washing the injury site with water and soap, sterilizing the wound and checking whether the patient has a blood-borne disease were done by 82.5, 89.5 and 68.9% of the respondents. Only 40.9% of the respondents had completed the three doses of HBV vaccination, and 27.3% didn’t receive any dose of the vaccine (Table 2).

### Association of demographic characteristics and KAP

The relationship between demographic characteristics and KAP groups is shown in Table 3. Age, occupation, educational degree, and work experience were not significantly associated with knowledge of HBV infection. A significant difference was found between the place of working (hospital) and the level of knowledge of HBV infection (X^2^ = 8.042, *p* = 0.005).

**Table 3:** Factors Associated with HBV knowledge attitude and practice

Demographic variables	No.	Average knowledge	*p*-value	Favourable attitude	*p*-value	Safe practice	*p*-value
Age group							
21–30 year	72	37(51.4%)	0.919	31(43.1%)	0.298	34 (47.2%)	0.576
31–40 year	12	6 (50.0%)		2 (16.7%)		7 (58.3%)	
41–50 year	13	8 (61.5%)		4 (30.8%)		6 (46.2%)	
More than 50	13	7 (53.8%)		4 (30.8%)		4 (30.8%)	
Hospital							
Saudi	64	30 (46.9%)	0.005	58 (90.5%)	0.124	20 (31.4%)	0.391
Saad Abul-Ella	46	34 (73.9%)		37 (80.4%)		18 (39.1%)	
Occupation							
Nurse	80	48 (60.0%)	0.528	69 (86.3%)	0.955	25 (31.3%)	0.235
Midwife	30	16 (53.3%)		26 (86.6%)		13 (43.3%)	
Education							
Below university	30	16 (53.3%)	0.528	24 (80.0%)	0.234	13 (43.3%)	0.235
University or above	80	48 (60.0%)		71 (88.8%)		25 (31.3%)	
Experience							
< 2 years	65	38 (58.5%)	0.943	57 (88.7%)	0.626	19 (29.2%)	0.159
> 2 years	45	26 (57.8%)		38 (84.4%)		19 (42.2%)	

## Discussion

This study examined the KAP towards HBV among nurses and midwives in two public hospitals specialized in obstetrics and gynaecology services in Khartoum, Sudan. The results of the study showed that 58.2 and 65.5% of the participants had an average level of knowledge and safe practice, while the majority of the participants had a favourable attitude towards the preventive measures of the HBV infection. These results indicate that there is a need for more HBV health promotion, targeted education, and training of nurses and midwives. Other studies reported that the level of the knowledge of hepatitis is low among different populations, including HCW, in several areas worldwide [23–25].

The findings of the study posited that the level of knowledge was not significantly associated with age, occupation, marital status, educational level, or working experience in this study. Another study conducted among HCWs in White Nile state in Sudan, on the contrary, showed that the level of knowledge was significantly associated with occupation and educational degree [26]. The differences may be attributed to the inclusion of a broader range of professions in the afore-mentioned study conducted in the White Nile state in Sudan, which considered all HCWs occupations, compared to this study which focused on nurses and midwives. According to findings, workplace (hospital) was the only demographic variable which is associated with the level of knowledge which may be attributed to the differences between these two hospitals. There was a better level of knowledge in Saad Abu-Ella hospital, which is a teaching university hospital, and it is a semi-private hospital. Other studies have identified several factors associated with the level of knowledge such as sufficiency of the educational programs in the hospital setting, vaccination status, and having family members or friends with active hepatitis [23, 27, 28].

The study findings postulated that half of the participants had a knowledge gap on PEP, which is an effective intervention for prevention of HBV transmission, this fact alerts that the knowledge of PEP for HBV in this study area needs improvement. Also, another striking finding of the study depicted that while near half of the participants have been exposed to HBV risky conditions (51.8%), the HBV vaccination coverage rate and level of knowledge about PEP were low. Such a situation is alarming and emphasizes the need to increase the level of awareness regarding blood born infections, including HBV infection.

More strategies should be implemented to prevent workplace exposure to reduce the risk of occupational exposure among HCWs in these, and to improve the level of knowledge and practice of PEP and vaccination coverage in healthcare facilities. In these settings, healthcare organizations should review their preventive strategies, which include written protocols for prompt reporting, evaluation, counselling, and treatment of occupational exposures that might place HCWs at risk for acquiring the infection and establish these systems if they aren’t already in place.

Following procedures should be performed by the healthcare organizations: implementation of standard precautions, availability of HBV vaccination, availability of enough gloves and safety boxes, and access to clinicians who can provide PEP during all working hours [29, 30]. In addition, HCWs should be trained in the prevention of blood-borne infections, including the need for vaccination against HBV. HCWs should be familiarized with the principles of post exposure management as part of job orientation and on-going job training [29, 30].

The findings of the study revealed a high rate of NSI (51%). Other studies conducted in Sudan, Kenya, Iran, and India revealed similar high NSI rates among HCWs [3, 31–33]. In developing countries, the severity of such issues tends to be underestimated because many HCWs do not report exposure to hepatitis B virus infection or the risk of exposure. This could compromise appropriate post exposure management [34].

Although a safe and effective vaccine against HBV is available throughout the world, HCWs in developing countries still remain at risk because most of the participants are not vaccinated against HBV [13]. HBV vaccination coverage rates for HCWs range from 18% in Africa to 77% in Australia and New Zealand [35]. In this study, HBV vaccination coverage rate was higher than in another study conducted in Ethiopia where the vaccination schedule has been recently completed (5.4%) [36]. That study conducted in Ethiopia reported that low vaccination status was explained by non-availability of the vaccines and the cost issues [36]. Other alternative preventive measures such as wearing gloves and hand hygiene are simple and cost-effective; however, still require staff accountability and behavioural change.

The findings of this study need to be considered in the context of some limitations; this is a cross-sectional study done in two sites, which might limit results generalization for all settings in the country. Further analysis is needed to investigate risk factors that influence KAP level of HCWs towards HBV infections.

## Conclusion

It can be concluded that most of the nurses and midwives working at Saudi and Saad Abul-Eleella hospitals are aware of HBV infection. However, a significant proportion of the participants lack the necessary knowledge about post exposure management and prevention of occupational exposures. The study reveals a low level of HBV vaccination coverage rate and high rate of NSI. Further strategies for preventing workplace exposure, training programs on HBV infection, including PEP, and increasing vaccination coverage rate of all HCWS are highly recommended.

## Supplementary information

**Additional file 1.** Interview guide: the structured questionnaire used for this study.

## Data Availability

The datasets used during the current study are available from the corresponding author on reasonable request.

## Abbreviations

HBsAg: Hepatitis B surface antigen
HBV: Hepatitis B virus
HCWS: Healthcare workers
IG: Immunoglobulins
KAP: Knowledge, attitude and practice
MTCT: Mother to child transmission
NSI: Needle stick injury
PEP: Post exposure prophylaxis
