# Supplementary material for: Knowledge, attitude, and practice towards Hepatitis B infection among nurses and midwives in two maternity hospitals in Khartoum, Sudan
Source: BMC Public Health. 2019 Nov 29;19:1597. doi: 10.1186/s12889-019-7982-8 (PMC6884767; doi:10.1186/s12889-019-7982-8)
Supplement: Supplementary file 1 — Additional file 1. Interview guide: the structured questionnaire used for this study. [file 12889_2019_7982_MOESM1_ESM.docx]

Department of community medicine

Faculty of medicine

University of Khartoum

Questionnaire number

1. Age
2. Marital status: 1. married 2. Divorced 3. Widowed 4. single
3. Level of education: 1. Primary 2. secondary

3. University 4. Postgraduate

1. Occupation: 1. Nurse 1. Midwife
2. Duration of work as health care worker in this hospital:
3. Hospital: 1. Saudi 2.Saad Abul-Ella

----------------------------------------

1. Do you know there are several types of hepatitis?

1. Yes 2. No

1. Hepatitis B is a…….. Disease:

1. Bacterial 2. Viral 3. Parasitic 4. Don’t know

1. Hepatitis B infection may lead to: (you can chose more than one answer) :
2. Acute hepatitis
3. Chronic hepatitis
4. Liver cirrhosis
5. Liver cancer (hepatocellular carcinoma)
6. Death
7. Peptic ulcer
8. Don’t know
9. People can get hepatitis B infection through: (you can chose more than one answer) :
10. Blood transfusion
11. Sexual intercourse
12. Birth “from mother to child during birth”
13. Direct contact to any body fluids
14. Needle stick injury
15. Air
16. Don’t know
17. post-exposure prophylaxis of HBV infection is:

1. Washing and sterilization of wound

2. Immunoglobulin

3. Vaccine and immunoglobulin

4. Don’t know

----------------------------------------

1. Do you believe that instrument sterilization is important to prevent transmission?

1. Yes 2.No

1. Do you believe that wearing gloves wearing gloves is important to prevent transmission?
2. Yes 2. No
3. Do you believe that vaccination could prevent transmission?

1. Yes 2. No

1. Do you recommend PEP for those who had been exposed to HBV?

1. Yes 2. No

-----------------------------------------

1. Do you Sterilize instruments?
2. Always 2. Often 3.Never
3. Do you wear gloves during working
4. Always 2. Often 3. Never
5. Have you experienced a needle stick or sharps injury involving a needle or sharp instrument that had been used on a patient?
6. Yes 2. No
7. If your answer in Q18 is yes, what you did?

1. Wash with soup and water

2. Sterilize the wound

3. Check if the patient has a blood born disease

4. Nothing

1. Are you vaccinated against hepatitis B infection?
2. Yes 2. No
3. If vaccinated, how many doses?
4. One 2. Two 3. Three
